# Risk of coronavirus disease 2019 (COVID-19) from hospital admission during the pandemic

**DOI:** 10.1017/ice.2020.1249

**Published:** 2020-10-08

**Authors:** Krishna Nalleballe, Suman Siddamreddy, Sukanthi Kovvuru, Poornachand Veerapaneni, Bhaskar Roy, Sanjeeva Reddy Onteddu

**Affiliations:** 1 Department of Neurology, University of Arkansas for Medical Sciences, Little Rock, Arkansas; 2 Department of Medicine, University of Arkansas for Medical Sciences/Baptist Health Program, North Little Rock, Arkansas; 3 Department of Neurology, Yale School of Medicine, New Haven, Connecticut

*To the Editor—*Since the onset of the coronavirus disease-2019 (COVID-19) pandemic, hospitals all over the world have treated an unprecedented number of patients with COVID-19. At the same time, hospitals have noticed a downward trend in hospital admissions for non–COVID-19–related illnesses including diseases that need time-sensitive treatments like strokes and acute coronary syndromes.^1–3^ Elective procedures and surgeries were deferred. One of the potential etiologies for the decrease in hospital admissions could be concerns of contracting COVID-19 in the hospitals. As hospitals gradually return to their routine, the risk of COVID-19 from hospital admissions needs to be examined to ensure patient safety. In this study, we examined the risk of acquiring COVID-19 in the patients admitted to the hospitals with non–COVID-19–related conditions.

Data were obtained from TriNetX, a global clinical research platform that collects de-identified patient data that are updated in real time from 45 healthcare organizations. “COVID-19 Research Network” in TriNetX is a large COVID-19 database, which is also being used by the Food and Drug Administration (FDA) Sentinel Operations Center at the Harvard Pilgrim Health Care Institute to monitor priority drugs used for the care of the hospitalized COVID-19 patients. We analyzed patients who were discharged from the hospital with non–COVID-19–related illnesses (using discharges codes and excluding lab confirmed COVID-19 patients) between January 20, 2020, and June 30, 2020, who later tested positive for COVID-19 by reverse-transcription polymerase chain reaction (RT-PCR) within 14 days of discharge. For comparison, we obtained the total number of COVID-19–positive patients in United States, from the Centers of Disease Control and Prevention (CDC)^4^ up to July 14, 2020, among the total population of the Unites States from the US Census Bureau. Our local institutional review board deemed this study to be ‘not human subject research’ using global deidentified COVID-19 research network data designated for research use (IRB no. 261137).^5^

In total, 101,533 patients were discharged from the hospitals with non–COVID-19–related illnesses between January 20, 2020, and June 30, 2020. Among them, 44 patients (0.043%) tested positive for COVID-19 by RT-PCR within 14 days of discharge (see Table 1 for demographics and comorbidities). The percentage of positive COVID-19 patients among the total US population was 1.0353% (3,416,428 of 329,986,480) as of July 14, 2020.The odds of contracting COVID-19 is 24.1 times higher in the general population compared to hospitalized patients (OR, 24.1; 95% CI, 17.9–32.4; *P* < .001).

**Table 1.** Baseline Demographics and Comorbidities of Patients with Hospital Discharge Between January 20, 2020, and June 30, 2020, and Patients Who Acquired COVID-19 After Hospital Discharge

Characteristic	Study Group (n = 101,533)	COVID-19 in Study Group (n = 44)	*P* Value
Age, median y ± SD	51.4 ± 23.9	62.5 ± 14.1	**.002**
Sex, female, no. (%)	51,505 (50.7)	23 (52.2)	.83
Race, no. (%)			
White	66,225 (65.2)	26 (59.1)	.39
Black	24,794 (24.4)	14 (31.8)	.25
% of patients with comorbid conditions			
Hypertensive	56.7	86.3	**<.001**
Diabetes mellitus	30.3	43.1	.064
Cnronic kidney disease	22.0	40.9	.002
Ischemic heart	28.1	38.6	.122
Congestive heart failure	23.9	36.3	.053
Overweight	23.8	31.8	.214
Chronic obstructive pulmonary disease	15.9	29.5	.013
Asthma	13.2	25.0	.021
Atrial fibrillation	16.5	22.7	.269
Smoking	22.1	22.7	.921

Overall, this result suggests a low risk of COVID-19 from hospitalization. The proper use of PPE and appropriate precautionary measures may have helped reduce the risk of transmission of COVID-19 during hospitalization. Despite addressing a relevant question, our study has several limitations. Some SARS-CoV-2–positive patients may have contracted the virus from another source. Moreover, we did not examine the risk of COVID-19 in the outpatient setting. Nevertheless, this result is reassuring and should encourage timely treatment of healthcare problems, even during the COVID-19 pandemic, to avoid unnecessary complications.
